# Fertility preservation in women affected by gynaecological cancer: the importance of an integrated gynaecological and psychological approach

**DOI:** 10.3332/ecancer.2020.1035

**Published:** 2020-05-06

**Authors:** Valentina Lucia La Rosa, Simone Garzon, Giuseppe Gullo, Michele Fichera, Giovanni Sisti, Pasquale Gallo, Gaetano Riemma, Antonio Schiattarella

**Affiliations:** 1 Unit of Psychodiagnostics and Clinical Psychology, University of Catania, Catania, Italy; 2 Department of Obstetrics and Gynaecology, “Filippo Del Ponte” Hospital, University of Insubria, Varese, Italy; 3 Department of Obstetrics and Gynaecology, AOOR Villa Sofia Cervello, IVF Public Center, Palermo, Italy; 4 Department of General Surgery and Medical Surgical Specialties, University of Catania, Catania, Italy; 5 Lincoln Medical and Mental Health Center, 234 East 149 Street, 5th Floor, Bronx, NY 10451, USA; 4 Obstetrics and Gynaecology Unit, “Santa Maria delle Grazie” Hospital, Pozzuoli, Naples, Italy; 7 Department of Woman, Child and General and Specialised Surgery, University of Campania “Luigi Vanvitelli”, Naples, Italy

**Keywords:** fertility preservation, reproduction, quality of life, gynecological cancer, endometrial cancer, cervical cancer, ovarian cancer, fertility-sparing surgery

## Abstract

Gynaecological cancer treatment significantly affects the fertility of women in reproductive age. Surgery, chemotherapy and radiotherapy are the mainstays of ovarian, cervical and endometrial cancers and anatomically or functionally impact the uterus and ovaries. Moreover, the sexual function and psychological wellbeing of patients are highly weakened after a cancer diagnosis: depression, anxiety and impairment of quality of life represent a relevant concern for patient care. The potential loss of fertility could be more distressing than cancer itself. For this reason, it is of paramount importance to try to preserve fertility in women affected by gynaecological cancers. Recently, tailored fertility preservation therapies have been developed to meet the childbearing demand from more than half of women between 18 and 40 years with a diagnosis of cancer. Currently, fertility preservation techniques play a significant role in improving the quality of life of women with gynaecological cancer. In this scenario, we propose a narrative overview of the recent literature about the importance of a multidisciplinary approach in the management of fertility preservation in the case of gynaecological cancers.

## Introduction

The annual global cancer burden is estimated to reach about 18 million cases and more than 9 million deaths [1, 2]. Gynaecological cancers have a prevalence of 15%–20% of the total neoplasms involving women. The most common is uterine cancer (53%) [3–5], followed by ovarian (25%) [6, 7], cervical (14%) [8–10], vaginal and vulvar cancers [11–13], with more rare neoplastic forms, such as trophoblastic tumours [14]. About 20% of gynaecological cancer affects women under 40 years of age, who often have not completed parity or are before their first pregnancy [15–20]. In these patients, fertility preservation represents a unique point of paramount importance. Surgical therapy is demolitive for the reproductive organs in the majority of cases, and chemotherapy and radiotherapy affect the function of the uterus and ovaries if preserved. For these reasons, recent guidelines recommend that although the initial focus is on the cancer diagnosis, healthcare providers should discuss with their patients the impact of cancer treatments on fertility from the earliest stages of therapy, in order to guide women in choosing between different fertility preservation options [21–23].

In addition to the loss of function, the psychological consequences can sometimes be more significant. Sexuality and couple relationships can be severely affected in these patients, especially because gynaecological cancer has a strong impact on the female identity and threatens their feelings about their sexuality [24, 25]. Recent studies support the idea that infertility has implications that go beyond the immediate reproductive needs of patients [24, 25]. Many of these women greatly benefit from working on crucial psychosexual issues within a psychotherapeutic setting [26]. According to these considerations, it is evident that fertility preservation has fundamental importance for these women. Indeed, the literature about this topic confirms that patients think about fertility counselling as an essential issue in their care pathway. Moreover, adequate fertility preservation counselling allows the reduction of feelings of anxiety and worry related to fertility and improvement of health-related quality of life significantly [27, 28].

In this scenario, the purpose of this mini-review is to provide an overview of the impact of gynaecological cancer on women in reproductive age, deepening the importance of a multidisciplinary approach to fertility preservation.

## The impact of cancer and fertility preservation on quality of life

The prevalence of cancer diagnosis at childbearing age is increasing, while mortality continues to reduce [29]. This aspect presents various causes, such as primary prevention, widespread screening and improvement of diagnostic tools and treatment efficacy [1, 30]. New shreds of evidence report that about 75% of these reproductive-aged women diagnosed with cancer refer childbearing wishes [27, 31]; therefore, new priorities need to be set.

The diagnosis of gynaecological cancer represents a hard-emotional impact on women. Young women with gynaecological cancer experience the loss of menstruation and fertility with particular anxiety and stress. In particular, it has been underlined that psychological distress related to infertility is more pronounced in women who have not yet created their families and would still like to do so [24, 25, 32]. Furthermore, the literature on the topic reports a significant presence of anxiety, depression, low self-esteem, anger, irrational beliefs about cancer, suicidal thoughts and sleep disorders in women with gynaecological cancer [27, 31–33].

Sexual dysfunctions are one of the most frequent side effects of cancer therapies. Their prevalence in women surviving gynaecological cancer is estimated at 40% to 100% compared with about 19% to 50% of the healthy female population [34, 35]. Indeed, cancer can cause profound changes in the body image, especially in the cases of premature infertility or surgical disfigurement. In turn, a negative body image is associated with decreased sexual functioning and increased discomfort during intercourse [36]. Furthermore, all major cancer treatments (surgery, radiotherapy, chemotherapy and hormonal therapy) are associated with a significant impairment of sexual function [37]. According to the American Cancer Society, the most frequent female sexual problems caused by cancer treatments include vaginal dryness, low sexual desire, dyspareunia and difficulty in reaching orgasm [36, 38].

Several studies have investigated the impact of reproductive cancers on sexual satisfaction and needs of women of reproductive age. Mütsch *et al* [39] investigated sexual function and the needs related to the sexual sphere of adolescents and young women affected by both reproductive and other types of cancer. According to their results, sexuality is significantly worse in women with tumours affecting the reproductive sphere. More specifically, the Authors reported a significant deterioration of sexual satisfaction, changes in sexuality and increased sexual supportive care needs [39]. Similar data were highlighted in the study by Geue *et al* [40], which investigated couple relationships and sexuality in adolescents and young adults with cancer. According to the Authors, these patients reported high satisfaction with their relationships but, at the same time, experienced sexual problems and need support in dealing with sexual problems [40].

The awareness of having cancer implies a real regret for the perception of oneself, one’s health, one's identity as a woman and mother, as well as one's wellbeing in general [41]. The life of a woman with gynaecological cancer is, therefore, severely impaired, as well as the idea of death changes the prospects and life priorities [42, 43]. Also, the perception of time changes: the future seems to be threatening and the present is distressing [33, 42]. The self-image is essentially modified, and there is a decrease in psychological, sexual and social well-being. Personal identity is impaired, and the ability to cope with stress and planning can be compromised [13, 44, 45]. In this scenario, pregnancy and motherhood also take on an important symbolic value which should not be overlooked. Indeed, for a woman who survives cancer, motherhood is of fundamental importance becoming a mother is the symbol of hope in the future and of the life that wins over death [28, 46]. Consequently, the opportunity of achieving a pregnancy and having a child after a malignancy significantly improves the therapeutic process: in fact, the woman is more motivated to accurately follow the therapy and faces it with greater tranquility [27, 47–49]. For this reason, the possibility of procreating after cancer might be interpreted as an essential element of protection [48, 50, 51].

For all these reasons, fertility preservation techniques are important to improve quality of life of women with gynaecological cancer [28]. In this regard, it is mandatory to strictly follow the progression of cancer; however, it is equally crucial to improve as much as possible the patient’s psychosexual wellbeing, her quality of life and psychosocial functioning, due to the fact that these aspects affect both the compliance as well as the effectiveness of the therapy itself [17, 25, 52].

Nevertheless, proper information and expert counselling should focus on propose a unique plan for the single patient, taking into account her life perspective that can be acquired on active comprehension and listening of her needs, desires, fears and doubts. Furthermore, it is also important to provide an adequate sexual counselling, especially about decreased libido, inability to achieve orgasm, dyspareunia, vaginal dryness or loss of sexual pleasure [36]. However, it has been shown that clinicians rarely address psychological and sexual issues with gynaecological cancer patients. This difficulty can be explained by the lack of adequate knowledge and communication skills on the topic [53]. At the same time, the woman may avoid confronting these topics with the clinician because of a sense of embarrassment. As a consequence, the woman is most at risk of developing anxiety and depression [36]. For these reasons, it is very important that the health care professional has basic knowledge on the impact of cancer and its treatments on the patient's psychological and sexual sphere in order to improve communication as well as the adherence to the proposed treatments [28]. For this purpose, the involvement of a psychologist in the multidisciplinary team deeply reduces the level of stress, anxiety, and depression. Moreover, it allows a better adherence to therapy, reducing the negative impact on women’s quality of life [12, 59, 60].

## The importance of counselling for fertility preservation

It has been widely demonstrated that the different options for preserving fertility in women with gynaecological cancer are effective in improving the quality of life of these patients [47, 54]. Different studies confirmed the positive association between the proposal on fertility preservation treatments and the quality of life improvement of cancer survivors [51, 52]. Based on this purpose, a fertility preservation counselling can be offered at all women, including those in pre- and post-pubertal age [55, 56]. In recent years, several guidelines and clinical recommendations concerning fertility preservation techniques have been developed. They strongly recommend that patients should be informed on available fertility preservation options before starting anticancer treatments [22, 57]. However, limited information about these treatments still exists, and women are quite often uninformed about choices and possibilities [47]. In addition, the physician should acknowledge that it is mandatory to give advices that should be related to reproductive prognosis and the risk of treatment for infertility [22, 47, 58]. Other important aspects should be considered in order to provide the best fertility preservation advices. The patient's decision on fertility conservation are also impacted by financial resources, prejudices, lack of adequate support, anxiety and doubts about treatments [59]. For this reason, only the knowledge of an expert physician could offer adequate information and gives the possibility of listening to the women’s suffering and doubts [58, 60, 61].

Concerning this point, some studies have investigated oncologists’ practice and attitude about fertility preservation. Some of them underlined that almost one-third of oncologists are not able to explain fertility issues [62]. Although so, most physicians offer high visibility to the problem and provide information about fertility preservation options [61, 63]. Physicians’ difficulty in talking about preserving fertility is generally due to their insufficient knowledge regarding these techniques as well as to the lack of specific communication strategies [47, 64]. In this regard, the communication between patient and physician about fertility preservation may be influenced by several factors such as physician knowledge of fertility preservation techniques and their efficacy, patient’s anxiety and stress and economic problems [65]. Other factors may also affect the effectiveness of fertility preservation counselling. For example, cognitive aspects, such as biases and heuristics, may make decision-making about fertility preservation more difficult and less aware [59]. In these cases, decision support interventions or decision aids can be useful to reduce possible biases in the decision-making process [59]. More specifically, decision aids provide information about cancer and female fertility, discussing the different available fertility options and the advantages and disadvantages of each one [68]. This type of approach is very useful in increasing knowledge and reducing decisional conflict without increasing anxiety. The P5 approach can help to improve decision-making about fertility preservation, transforming women into active decision-makers in the treatment process [67].

For all these reasons, it is important that reproductive counselling is carried out by a multidisciplinary team that also includes a psychologist [27, 47]. Indeed, the new concept of oncofertility represents a new crucial challenge in the treatment and assistance of cancer patients. In this regard, the Oncofertility Consortium can be considered an example of an adequate multidisciplinary approach for fertility preservation that includes oncology, reproductive medicine and public health [68, 69].

## Conclusion

Fertility preservation techniques can be effective in the improvement of the quality of life of women with gynaecological cancers. The clinical experience and the international literature about the topic deepen the importance of the role of quality of life on psychological outcomes [48]. The objective of a better life for the cancer survivor and the hope of becoming a mother represent a fundamental aspect of improving self-esteem. Future studies are necessary to develop effective therapeutic counselling approaches to be combined with oncological therapies with the aim of reducing the psychological impact of the cancer experience and improving the quality of life for women.

## Conflicts of interest

The authors declare that they have no conflicts of interest.

## Funding statement

No funding was obtained for this study.
